# Seroprevalence of vector-borne pathogens and molecular detection of *Borrelia afzelii* in military dogs from Portugal

**DOI:** 10.1186/s13071-016-1509-2

**Published:** 2016-05-10

**Authors:** Ana Margarida Alho, Joana Pita, Ana Amaro, Fátima Amaro, Manuela Schnyder, Felix Grimm, Ana Cristina Custódio, Luís Cardoso, Peter Deplazes, Luís Madeira de Carvalho

**Affiliations:** CIISA, Faculty of Veterinary Medicine, Universidade de Lisboa (ULisboa), Lisbon, Portugal; Portuguese Air Force, Lisbon, Portugal; National Institute for Agrarian and Veterinary Research (INIAV, I.P.), Lisbon, Portugal; Centre for Vectors and Infectious Diseases Research, National Institute of Health Ricardo Jorge, Águas de Moura, Portugal; Institute of Parasitology, Vetsuisse Faculty, University of Zürich, Zürich, Switzerland; Department of Veterinary Sciences, School of Agrarian and Veterinary Sciences, University of Trás-os-Montes e Alto Douro (UTAD), Vila Real, Portugal

**Keywords:** Canine vector-borne diseases, Dog, Epidemiology, Military, Seroprevalence, Portugal, *Borrelia afzelii*, *Borrelia burgdorferi* (*sensu lato*), Toscana virus, Zoonosis

## Abstract

**Background:**

Canine vector-borne diseases (CVBDs) are increasingly being reported worldwide and represent a serious threat to both animal and public health. Military dogs may constitute a risk group for the agents causing these diseases, as they frequently work outdoors in different areas and are thus exposed to vector arthropods. In order to assess the risk of exposure of this type of dogs, a serological and molecular survey was conducted in military working dogs in Portugal. One hundred apparently healthy dogs were surveyed. Serum samples were tested for antigens of *Angiostrongylus vasorum* and *Dirofilaria immitis*; and for antibodies to *A. vasorum*, *Anaplasma* spp., *Babesia* spp., *Ehrlichia canis*, *Leishmania infantum*, *Rickettsia* spp. and Toscana virus. Serum was tested by polymerase chain reaction for *Borrelia burgdorferi* (*sensu lato*), with sequencing of the DNA products.

**Results:**

Forty-nine per cent of the dogs were seropositive for antibodies against *Rickettsia* spp.*,* 16 % for *Anaplasma* spp., 13 % for *L. infantum*, 7 % for *E. canis*, 5 % for *A. vasorum* (including 1 % positive for both antibodies and circulating antigens), 3 % for *Babesia* spp. and 1 % positive for Toscana virus. *B. burgdorferi* (*s.l.*) was detected in eight out of 94 dogs tested (8.5 %) and in three cases (3.2 %) nucleotide sequence analysis showed identity with the genospecies *Borrelia afzelii*. No positive cases were recorded for *D. immitis.* Overall, 66 % of the dogs were positive for at least one out of the eight tested CVBD agents, six of which are zoonotic (i.e. *Anaplasma* spp., *Borrelia* spp., *E. canis*, *L. infantum*, *Rickettsia* spp. and Toscana virus)*.* Serological specific antibody detection against more than one CVBD agent (including molecular detection of *Borrelia* spp.) was recorded in 25 % of the dogs, comprising 19 % with positive reaction to two agents, 5 % to three agents and 1 % to four agents.

**Conclusions:**

These results reveal a high occurrence of CVBD agents in military working dogs in Portugal and highlight the need to maintain a comprehensive and regular prophylaxis to reduce the contact between working dogs and those pathogens. For the first time in Portugal, *B. afzelii* DNA was identified in dogs and a dog was found seropositive for antibodies against Toscana virus.

## Background

Canine vector-borne diseases (CVBDs) are an emerging problem worldwide due to their frequency, morbidity and zoonotic potential, representing a serious threat to animal and public health [1]. These diseases are caused by a wide range of pathogens, comprising viruses, bacteria, protozoa and helminths transmitted to dogs by different arthropods, namely ticks, fleas, mosquitoes and phlebotomine sand flies [2]. Several factors have been linked to the expansion of CVBDs, including an increased exposure to old and new infectious agents. Enhanced international commerce, faster and incremented global transport, deforestation and urbanization, abundance of wildlife hosts, demographic and political changes, climate alterations and drug resistance among vectors and pathogens, are making the spread of ectoparasites and their pathogens a no-boundary global event [3].

Bacterial agents of CVBDs such as *Anaplasma platys* (the cause of infectious canine cyclic thrombocytopenia), *Anaplasma phagocytophilum* (granulocytic anaplasmosis), *Borrelia burgdorferi* (*sensu lato*) complex (Lyme disease), *Ehrlichia canis* (canine monocytic ehrlichiosis) and *Rickettsia conorii* (Mediterranean spotted fever) are tick-borne diseases of increasing concern [4–6]. Also important are some protozoal agents of CVBDs including *Babesia canis* and *Babesia vogeli* (canine babesiosis or piroplasmosis), also vectored by ticks [7], and *Leishmania infantum* (leishmaniosis), vectored by phlebotomine sand flies [8]. Other relevant CVBD agents are the nematode *Dirofilaria immitis*, a mosquito-borne pathogen, which induces cardiopulmonary dirofilariosis or heartworm disease; and Toscana virus, which is an arbovirus (i.e. an arthropod-borne virus) vectored by phlebotomine sand flies [9]. Although not transmitted by arthropods but by slugs or snails, the nematode *Angiostrongylus vasorum*, also known as the “French heartworm” (causing canine angiostrongylosis), is an increasingly reported pathogen in Europe [10]. In general, canine infections with CVBD agents range from mild to severe and life-threatening forms. Clinical signs may include lethargy, weight loss, fever, lymphadenomegaly, poor appetite or anorexia, but are often variable and non-specific, thus requiring diagnosis to be complemented at the laboratory level. In addition, the vast majority of these miscellaneous pathogens (i.e. *Anaplasma* spp., *Borrelia* spp., *E. canis*, *L. infantum*, *R. conorii* and Toscana virus) have also zoonotic character, causing disease in humans, thus representing a great veterinary and public health threat.

Military working dogs (MWD), also known as police dogs, are specifically trained to assist security and law-enforcement personnel in their work. These animals make periodic fieldwork in the most diverse climatic conditions of national and international territories, spending long periods outdoors, which increase the contact with wild animals and diverse types of vectors. The nature of their activities exposes them to risk factors distinct from those of common pet dogs, and may make them more susceptible to CVBDs [11]. Likewise, MWD have intense contact with people, as they are paired with a dog handler, i.e. someone who trains and is accompanied by the animal for long periods, a fact that increases the risk of transmission of zoonotic pathogens.

Little is known about the risk of MWD regarding CVBDs. Few studies have been conducted so far and no surveillance mechanisms are in place to assess prevalence or geographical range in Portugal and Europe [12]. Considering the emergence of CVBDs in Europe, as well as the lack of studies regarding CVBDs in MWD, an epidemiological study was conducted, involving serological and molecular testing of dogs, kept in military bases across continental and insular Portugal.

## Methods

**Fig. 1:**
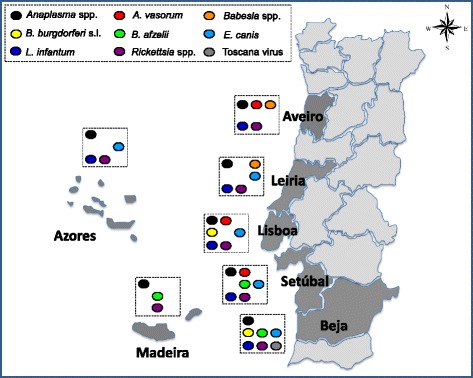
Regional occurrence (presence or absence) of vector-borne pathogens and *A. vasorum* in military working dogs from the seven air bases in mainland Portugal (Aveiro, Beja, Leiria, Lisboa and Setúbal) and on the Atlantic archipelagos of Azores and Madeira

A survey was conducted with 100 MWD belonging to the Portuguese Air Force. Blood was collected in distinct air bases in mainland Portugal (districts of Aveiro, Beja, Leiria, Lisboa and Setúbal) and also on the Atlantic archipelagos of the Azores and Madeira (Fig. 1). Serum was obtained from each dog and stored at -20 °C until use.

A complete record was kept for each sampled dog, including gender, age, breed and body condition. All dogs were apparently healthy with no clinical signs or historical abnormalities compatible with CVBDs. The dogs were housed outdoors. All animals received a combination tablet of praziquantel, pyrantel pamoate and febantel every four months (Drontal® Plus XL, Bayer Animal Health); a deltamethrin-impregnated collar every four months (Scalibor®, MSD Animal Health); an ivermectin tablet monthly (Heartgard®, Merial); and an imidacloprid and permethrin spot-on monthly (Advantix®, Bayer Animal Health).

Out of the 100 dogs tested, there were 92 males and 8 females. Age ranged from 7 to 132 months (median: 60 months) and average body condition was 4.9 (range: 1 to 9). Six breeds were represented: German Shepherd (*n* = 64), Labrador Retriever (*n* = 15), Belgian Shepherd (*n* = 16), Dutch Shepherd (*n* = 3), Rottweiler (*n* = 1) and Dobermann (*n* = 1).

To test for the presence of *D. immitis* circulating antigens, a rapid commercial qualitative antigen kit WITNESS® Dirofilaria (Synbiotics, Europe) was used. All procedures were performed as recommended by the manufacturer. Sera were tested by enzyme-linked immunosorbent assay (ELISA) for detection of specific antibodies to *L. infantum*, as described by Mettler et al. [13]. Sandwich ELISAs were used for detecting antibodies to *A. vasorum* [14] and for the presence of *A. vasorum* circulating antigens [15]. Commercial immunofluorescent antibody tests (IFAT) were used to detect the IgG-antibodies to *Anaplasma* spp. (MegaScreen® FLUOANAPLASMA ph. kit), to *Babesia* spp. (MegaScreen® FLUOBABESIA canis kit), to *E. canis* (MegaScreen® FLUOEHRLICHIA c. kit) and to *Rickettsia* spp. (MegaScreen® FLUORICKETTSIA con. kit), according to the manufacturer’s instructions (Megacor, Horbranz, Austria). Sera were tested for immunoglobulin G (IgG)-class antibodies against Toscana virus using an in-house IFAT; and samples with IgG titres of 32 were considered as positive.

For molecular detection of *B. burgdorferi* (*s.l.*), DNA extraction from serum samples was carried out using the QIAamp DNA Blood Mini Kit (Qiagen, Valencia, CA, USA) tissue protocol, with some modifications including a mechanical lysis and the addition of carrier DNA (Salmon Sperm DNA [Sigma, St. Louis, MO, USA] at a final concentration of 10 μg/μl). Sera were screened for the presence of *B. burgdorferi* (*s.l.*) DNA using a nested PCR targeting the 5S-23S (rrf-rrl) rRNA intergenic spacer region using the primers pairs (23SN1 and 23SC1 - outer primers; 23 N2 and 5SCB - inner primers), as described by Rijpkema et al. [16]. The amplification reactions were performed in a C1000 thermocycler (Bio-Rad, Hercules, CA, USA) with an initial step at 94 °C for 3 min, followed by 25 rounds of temperature cycling (94 °C for 30 s, 52 °C for 30 s, and 72 °C for 1 min) for outer primers and 40 rounds of temperature cycling (annealing step of 54 °C for 30 s) for inner primers and an ending step at 72 °C for 7 min for both amplification reactions. A DNA solution extracted from *B. burgdorferi* (*s.l.*) culture and ultrapure water were used as positive and negative controls of amplification, respectively. The PCR amplified products were analysed by 1.5 % agarose gel electrophoresis and DNA-positive samples were sequenced at StabVida (Caparica, Portugal) using internal PCR primers. Nucleotide sequence analysis and comparison with other relevant reference sequences were performed using the BLAST suite at GenBank®.

Whenever appropriate, the chi-square or Fisher’s exact tests were used to compare proportions, and a probability *P*-value < 0.05 was considered as statistically significant. Exact binomial 95 % confidence intervals (CI) were established for proportions. Analyses were done using the StatLib and SPSS® 20 software for Windows.

## Results

Forty-nine per cent of the dogs were seropositive for antibodies against *Rickettsia* spp.*,* 16 % for *Anaplasma* spp.*,* 13 % for *L. infantum,* 7 % for *E. canis,* 5 % for *A. vasorum* (including 1 % positive for both antibodies and antigens), 3 % for *Babesia* spp. and 1 % positive for Toscana virus. *B. burgdorferi* (*s.l.*) DNA was detected in eight out of 94 dogs tested (8.5 %) and in three cases (3.2 %) sequence analysis showed identity with the genospecies *Borrelia afzelii*. No positive results were recorded for *D. immitis* antigen*.* Overall, 66 % of the dogs were positive for at least one out of the eight tested agents of CVBD, six of which are of zoonotic concern (i.e. *Anaplasma* spp., *Borrelia* spp., *E. canis*, *L. infantum*, *Rickettsia* spp. and Toscana virus). In addition, single antibody positivity to *A. vasorum* was found in three dogs, accounting for an additional 3 % prevalence. Serological specific antibody detection against single CVBD agents (including molecular detection of *Borrelia* spp.) was recorded in 41 % of the dogs. Positive reactions to more than one CVBD agent (including *Borrelia* spp.) was recorded in 25 % of the dogs, comprising 19 % positive to two agents, 5 % to three agents and 1 % to four agents (Table 1). Positive animals were distributed over all sampled areas in the country and a southward trend of increased pathogen diversity was observed (Fig. 1). Sequences identical to *B. afzelii* were found in two dogs from Setúbal and Beja (GenBank® accession no. KU891495) and in a dog from Madeira (GenBank® accession no. KU891496). Sequencing results from the other samples were inconclusive due to low quality of the obtained sequences.

**Table 1:** Serological specific antibody detection against vector-borne pathogens (VBP) in military working dogs from Portugal (including molecular detection of *Borrelia* spp.)

Agent(s)	Positive dogs		
	*n*	%	CI %
Positive reaction against single VBP	41^a^	41.0^f,g,h^	31.3–51.3
*Anaplasma* spp.	5	5.0	1.6–11.3
*Borrelia burgdorferi* (*sensu lato*)	4^b^	4.0	1.1–9.9
*Ehrlichia canis*	1	1.0	0–5.4
*Leishmania infantum*	3	3.0	0.6–8.5
*Rickettsia* spp.	28^c,d^	28.0	19.5–37.9
Positive reaction against two VBP	19	19.0^f,i,j^	11.8–28.1
*Anaplasma* spp. + *B. burgdorferi* (*s.l.*)	1^b^	1.0	0–5.4
*Anaplasma* spp. + *E. canis*	2^c^	2.0	0.2–7.0
*Anaplasma* spp. + *Rickettsia* spp.	5	5.0	1.6–11.3
*Babesia* spp. + *Rickettsia* spp.	1	1.0	0–5.4
*B. burgdorferi* (*s.l.*) + *Rickettsia* spp.	2^b^	2.0	0.2–7.0
*E. canis* + *Rickettsia* spp.	2	2.0	0.2–7.0
*L. infantum* + *Rickettsia* spp.	6^e^	6.0	2.2–12.6
Positive reaction against three VBP	5	5.0^g.i^	1.6–11.3
*Anaplasma* spp. + *B. burgdorferi* (*s.l.*) + *E. canis*	1	1.0	0–5.4
*Anaplasma* spp. + *Rickettsia* spp. + *L. infantum*	1	1.0	0–5.4
*Anaplasma* spp. + *Rickettsia* spp. + Toscana virus	1	1.0	0–5.4
*Babesia* spp. + *L. infantum* + *Rickettsia* spp.	2^e^	2.0	0.2–7.0
Positive reaction against four VBP	1	1.0^h,j^	0–5.4
*Anaplasma* spp. + *E. canis* + *L. infantum* + *Rickettsia* spp.	1	1.0	0–5.4
Single + co-infections	66	66.0	55.8–75.2

^a^Including four dogs singly positive for *Angiostrongylus vasorum*

^b^including one DNA sequencing result of *Borrelia afzelii*

^c^including one dog positive for *A. vasorum*

^d,e^three and one dogs not tested for *B. burgdorferi* (*s.l.*), respectively

^f,g,h^ *P* < 0.001

^i,j^ *P* ≤ 0.002; CI: 95 % confidence interval

No statistically significant associations were found for positivity to CVBD agents among the gender and age categories.

## Discussion

Out of the tick-borne pathogens, *Rickettsia* spp. was the most prevalent, followed by *Anaplasma* spp*.*, *E. canis*, *Babesia* spp. and *B. burgdorferi* (*s.l.*) In addition, *Rickettsia* spp. and *Anaplasma* spp. were detected in all the areas assessed, either in mainland or on insular regions. Besides, *L. infantum-*positive dogs were distributed throughout all the regions of mainland, and also on the Atlantic archipelago of Azores. For the first time, *B. afzelii* DNA was detected in dogs in Portugal, with this being a genospecies usually associated with small mammals and one of the causative agents of the most common tick-borne diseases in Europe and North America. Additionally, one dog was found positive for antibodies to Toscana virus, indicating a previous exposure to this agent. Although to date there is no evidence that dogs can develop disease when infected with this virus, this cannot be excluded, as well as their potential as amplifying hosts in the Toscana virus cycle [9]. In addition, specific antibodies against *A. vasorum* were detected in 5 % of the MWD, including one case simultaneously positive for *A. vasorum* antigen, which denotes an on-going infection. These data bring new information concerning the *A. vasorum* presence and geographical distribution in Portugal, as only a few cases of infection are documented in dogs from Portugal [17, 18].

In the present study, a very high number of dogs were found to be positive for at least one pathogen, with two thirds of them being positive to at least one of the CVBD agents tested and/or *A. vasorum* (Table 1). In fact, co-infection is a frequent condition in dogs, since several arthropods are competent vectors of more than one pathogen and may share the same environment. This is the case of the brown dog tick, *Rhipicephalus sanguineus* (*sensu lato*), known for its worldwide distribution, which serves as confirmed vector for *E. canis* and *R. conorii,* and as presumed vector for *A. platys* [19].

Co-infection is frequent all over Portugal, as previously evidenced by Cardoso et al. [20], where 14 % of apparently healthy dogs and 46.3 % of clinically suspect dogs were seropositive to at least one tested agent out of *Anaplasma* spp., *B. burgdorferi* (*s.l.*), *D. immitis*, *E. canis* and *L. infantum*. Similar findings were evidenced by Menn et al. [21], in southern Portugal, where 87 % of autochthonous shelter dogs were positive to at least one of the following: *A. phagocytophilum*, *B. canis*, *E. canis*, *H. canis*, *L. infantum*, *R. conorii* and microfilariae. In fact, Portugal is a country where several CVBDs are endemic, a situation which is partially explained by the mild Mediterranean climate that favours vector development and survival, which in turn contributes to justify the high prevalence levels detected in the present study. Additionally, many of these CVBD agents are of zoonotic concern with dogs serving as potential reservoirs or sentinels for wide variety of human infections. In fact, the close physical contact and daily interaction between military dogs and their handlers may increase the potential risk for the transmission of zoonotic pathogens. This is the case of *Anaplasma* spp., *B. burgdorferi* (*s.l.*), *E. canis*, *L. infantum*, *Rickettsia* spp. and Toscana virus, among others.

The prevalence detected in the present study may also represent the reality of MWD from military forces in other countries as they occasionally perform missions abroad. Previous studies conducted in MWD are few and punctual, and have shown a wide variation on the prevalence of canine vector-borne infections, mainly depending on the area under study, the diagnostic methods used and the ongoing prophylactic regimen [12, 22]. In a serological study to assess the exposure of MWD to tick-borne pathogens in South Korea, seroprevalence for *Anaplasma* and *Ehrlichia* were 4.4 % and 0.6 % based on ELISA, and 24.7 % and 22.5 % based on IFAT, respectively, and 1.1 % for *B. burgdorferi* (*s.l.*) based only on ELISA [23]. In Spain (Madrid), out of 131 dogs from the National Police Department, 2.3 % had antibodies to *E. canis* [24]. In Slovakia, out of the 710 police and military dogs investigated for the presence of microfilariae in blood, 18 % were diagnosed positive for *Dirofilaria* infection [25]; in New Caledonia, where canine dirofilariosis is endemic, a serological study revealed no positive results for *D. immitis* antigen in a population of MWD undergoing moxidectin prophylaxis [26]. Regarding *L. infantum*, a serological study performed in three MWD kennels in southeastern France showed a seroprevalence of 11.6 % [11].

It is important to keep in mind that the positive serological results presented is this study might be due to either an on-going infection or simply to a previous contact or exposure to the agent. For that reason, and whenever available and economically feasible, serological screenings should be complemented with molecular-based detection methods to ascertain on whether infections are active or not [19]. Likewise, serological cross-reactivity could occur between pathogens and thus PCR would be an advantage to achieve an accurate etiological diagnosis and to establish which species are implicated and circulating in the population. Yet, it must be emphasized that despite the tight prophylactic regimen implemented in this MWD, exposure to multiple CVBD agents was observed among this canine population, suggesting they should be regarded as a risk group. In spite of that, no dog showed any clinical signs. This is quite relevant as they can act as “silent” reservoirs and sentinels, fostering the perpetuation and transmission of endemic or exotic pathogens among other animals. Furthermore, subclinically infected dogs can transport arthropods harbouring pathogens into close proximity to people or even serve as a “direct” reservoir for human vector-borne infections, as several of these CVBDs have a zoonotic impact [27]. Also considering the impact of these diseases on the health of dogs, it is thus crucial to increase knowledge concerning their epidemiological situation and ensure routine screening. The results herein presented are essential to a better understanding of the potential CVBDs in this peculiar population. These new data will be useful for both medical and veterinary services engaged in the control of vector-borne diseases under the scope of One Health, and will serve as a reference for future research, prevention and control actions.

## Conclusions

In terms of the tested pathogens, this is the most comprehensive study carried out to assess the exposure of MWD to agents of CVBDs worldwide, and presents the first report of a seropositive dog for Toscana virus in Portugal, as well as the first time *B. afzelii* DNA has been identified in dogs in the country. Although these animals have daily monitoring, balanced nutritional support, regular medical care, tight prophylaxis and anti-parasitic control, their activities seem to steadily increase their contact with CVBD agents. Taking into account their long periods of work outdoors (both day and night) and their high mobility, these dogs are at a high risk of exposure to vectors and of contact with other domestic and wild animals, thus acting as a sentinel population. Considering that many of these CVBD agents are of significant zoonotic concern, an integrated approach under the scope of “One World, One Health” should be put in practice to control pathogens and promote higher animal and public health standards. Further epidemiological studies are needed to improve scientific knowledge and risk assessment concerning MWD and CVBDs.

### Ethics approval

All the clinical procedures in this study were in accordance with Portuguese (Decree-Laws no. 314/2003 and no. 113/2013) and European legislation for the protection of animals.

### Consent for publication

Not applicable.
